# The epidemiology workforce: are we planning for the future?

**DOI:** 10.1186/1743-8462-6-26

**Published:** 2009-11-29

**Authors:** Alice R Rumbold, Catherine M Bennett

**Affiliations:** 1 Menzies School of Health Research, Charles Darwin University, Australia and the Disciplines of Obstetrics and Gynaecology & Public Health, The University of Adelaide, Australia; 2 Melbourne School of Population Health, The University of Melbourne, Australia; 3 School of Health and Social Development, Deakin University, Australia

## Abstract

Epidemiology has a central role in public health practice, education and research, and is arguably the only discipline unique to public health. A strong perception exists among epidemiologists in Australia that there is a substantial shortage in epidemiological capacity within the health workforce and health research, and that there are few graduates with sufficient high-level epidemiological training to fill the educational and leadership roles that will be essential to building this capacity. It was this concern that led the Australasian Epidemiological Association (AEA)--the peak professional body for epidemiologists in Australia and New Zealand--to convene a working group in 2007 to assess and address these concerns. This article summarises the key training challenges and opportunities discussed within this group, and the larger organisation, with the intention of stimulating greater public debate of these issues.

## Introduction

In Australia, epidemiology training has been brought to the forefront in recent years by several initiatives; foremost, the 2005 Public Health Education and Research Program (PHERP) Review. PHERP is an initiative of the Australian Government to support public health training and build workforce capacity in partnership with Australian academic institutions; it is scheduled to end in 2010. The PHERP Review report [1] identified a lack of critical mass of expertise in epidemiology, alongside other disciplines such as biostatistics, health economics and public health nutrition. Some main reasons for the epidemiology deficit were identified as being: (a) the growth in clinical epidemiology supporting evidence- based practice; (b) increased demand particularly from the private sector; (c) technology advances resulting in increased access to quality health information; and (d) the fact that epidemiology specialisations continue to make up a relatively small part of Master of Public Health (MPH) programs [1].

Subsequent to the PHERP Review, the National Quality Framework for Public Health Education and Research [2] and the Public Health Competencies project [3] set about developing competency standards for training in public health practice. This ongoing competency process aims to set national minimum standards for research capacity and professional practice. However, the focus has been on generalist core competencies within MPH degrees [3] rather than advanced discipline-specific theory and skills.

In conjunction with these initiatives, a series of discussions have occurred in successive workshops at annual scientific meetings (ASM) of the Australasian Epidemiological Association (AEA). Participants in these forums have included both public health education providers and 'end-users' of graduates, many of whom were senior AEA members. The first of these forums occurred in 2006, prompted by concern across the profession about the size and capacity of the epidemiological workforce. Among participants, there was a strong perception of a lack of 'higher-level' and 'research-capable' epidemiologists in Australia. Many senior researchers reported difficulties in identifying and appointing staff with appropriate skills. However, despite the strong shared perception, employers, researchers and practitioners had not been collecting evidence to confirm and quantify the skills gap.

We have been accumulating evidence from two subsequent workshops, in 2007 and 2008, held in conjunction with the AEA annual scientific meetings and an AEA working group established after the 2006 forum to define issues around the perceived epidemiology skills shortage in Australia. Therefore, we would add to the abovementioned barriers identified by PHERP [1] that there has been a lack of a clear and structured pathway into specialised epidemiology training. The purpose of this article is to summarise discussions held to date within these AEA forums, propose recommendations to improve the level of epidemiological expertise in Australia, and to invite comment from an audience broader than the AEA membership.

### Epidemiology training opportunities and challenges

Formal epidemiology training programs in Australia range from those with basic epidemiology as a core component (health professional degrees, generalist public health and professional doctoral degrees, service-based public health training schemes) to specialist epidemiology programs (dedicated masters' degrees or specialisations and research higher degrees with a substantial, advanced epidemiology component). While there has been a considerable expansion in the number of MPH programs and student places available in Australia in the past decade--partly supported through PHERP funding initiatives--few offer a strong specialisation in epidemiology. It is therefore predominantly the specialist coursework and research higher degrees that offer the level of advanced epidemiology training that will help to address a critical higher level skills shortage.

If we are to produce professionals with the potential to lead and teach the next generation of professional epidemiologists, then building the capacity of the epidemiology workforce will require approaches that ensure both the depth and comprehensiveness of theoretical understanding and skill among public health practitioners and academics. Achieving this will require strategies that increase the number of people who have the capacity and are willing to specialise in epidemiology. This could be achieved either through an educational pathway that leads to a research higher degree, or as part of ongoing professional development as a practitioner. Below we summarise some key opportunities and challenges around creating these pathways, identified in the AEA forums.

### Coursework training

Initial discussions within the working group identified the need to understand the specialist epidemiology training that exists, and the volume, characteristics and pathways of current students and graduates of these programs. Information available from a training survey of postgraduate training institutions conducted by the AEA in 1999 identified seven specialist epidemiology masters' degrees (other than MPH degrees) and five diploma-or certificate-level epidemiology qualifications [5]. A search of Australian university websites in 2007 revealed a further five masters-level specialised courses being available, and a substantial increase in certificate- and diploma-level epidemiology qualifications. The indication we have is that none of these programs is at capacity, so it is unclear what impediments might exist in terms of access to prospective students, whether early career or as part of continuing professional development. Program entry requirements, cost, and flexibility of delivery for working or remote students may be barriers, or it may be that we are simply failing to raise the profile of epidemiological careers to attract students to this kind of training in the first instance.

### Research training

In addition to creating pathways into specialised coursework degrees, it is equally if not more important to encourage suitable candidates into epidemiology research higher degrees. This may require defining what is meant by a 'PhD in epidemiology', and relies on access to suitably qualified epidemiologists to supervise this training and advise on creative strategies for attracting prospective students.

A PhD in epidemiology could be defined as any body of work that applies or contributes to the theoretical underpinnings and methodologies of epidemiology. The challenge in defining what constitutes a PhD in epidemiology is not so much one of scope, but one of depth. How advanced or extensive does the application or contribution need to be? Research projects increasingly may incorporate mixed methods and the epidemiological component may be quite minor; who then decides whether this can be counted as an epidemiological PhD or not?

What matters is not only how many epidemiological PhD graduates there are, but how many of these can and do contribute to the building of research capacity through research supervision. There are also senior applied epidemiologists without PhDs who make important contributions to research training. As more universities move to a research training model that includes supervisory panels or committees, more of this expertise could be harnessed. Equally, access to PhD-qualified and non-PhD senior epidemiologists who are not employed within academia will be essential if we are to grow a sufficient critical mass of early career researchers. Therefore, honorary or joint appointments and/or joint supervision partnerships between universities, government and industry are also important in realising the growth required in research supervision capacity. We acknowledge that these types of arrangements are already in place within some institutions.

But perhaps the real question is where these PhD students will come from. MPH programs produce the largest critical mass of graduates trained in epidemiology. While some graduates will have just core basic skills, others will have completed an epidemiology specialisation and can move into PhD programs. However, on the whole, progression from MPH to PhD remains relatively rare. This is perhaps not surprising as those attracted to and selected for MPH programs are seeking career transitions and/or advancement that normally do not extend to independent researcher status.

As for other epidemiological coursework programs, we not only have to think about opportunity costs and access issues associated with higher degree research training--including those related to financial security--but also the visibility of epidemiological career options that will attract prospective students to such training. There is limited exposure to epidemiological training within undergraduate or professional entry degrees, including the health professions. Furthermore, honours programs in epidemiology are not widely available and other equivalent undergraduate programs that could feed into epidemiology higher degree training are limited. It is perhaps not surprising, therefore, that we are not overwhelmed with strong student interest in progressing to a PhD.

### Conversion fellowships

The forums included discussion of other models for building the epidemiology workforce, such as internships and conversion fellowships for higher degree trained professionals from other theoretically and methodologically related fields. The aim would be to enable a transition of these PhD graduates into epidemiological roles where relevant skills could be further developed and transposed to the public health or medical research contexts. Potential target groups for such fellowships could include PhD graduates from biomedical science and statistics. Critical success factors for such a model include funding availability and the selection of institutions available to offer such fellowships and appropriate supervision. In addition, agreement would have to be reached on the critical skills, knowledge and attributes of conversion fellows--the formal training required to build these skills in graduates from non-health backgrounds.

### Integrating coursework into research higher degrees

The provision of formal epidemiology coursework within PhD programs was another feature of our discussions. At a minimum this was thought to have the potential to ease the transition from honours degrees to research higher degrees, particularly where the honours degree is outside public health, such as the biomedical sciences. However, there is the potential for limited breadth of knowledge, as theoretical understanding and applied skills developed during a PhD program in epidemiology may instill expertise limited by the scope of the project. For example, a graduate may be an expert in clinical trials but have little experience or knowledge of observational studies. Such graduates may not have sufficient knowledge or research experience to practise, conduct research, teach or supervise outside their specialty interest area.

A more fundamental concern is that PhD students are vulnerable to the lack of a solid methodological foundation. The North American Model addresses this through an embedded coursework curriculum in the doctoral program. Models in Australia take a similar approach without impinging on the size or standard of the dissertation. For example, the Melbourne School of Population Health at The University of Melbourne offers a PhD with coursework, and epidemiology students as well as PhD students across the medical and health sciences faculty can incorporate research methods subjects into their program, including the equivalent coursework to that required for a Master of Epidemiology.

Coursework embedded within PhDs not only provides efficient training in the theory and methods directly relevant to the thesis, but extends beyond that and enables PhD graduates to have a firm career foundation that is not project-specific. However, we acknowledge that many PhD programs currently have informal arrangements which allow students to undertake coursework public health subjects and that any move to increase formal coursework requirements will need to ensure the gains for the student are offset by any potential impact on candidature load and completion time.

### Specialist short courses

Increasing the number of advanced epidemiology short courses was also identified as being a means of increasing access to specialisation for higher degree students and already qualified professionals. This would also provide a professional development framework for building on basic skills acquired through work experience, or, for example, in the MPH. This will in part rely on a critical mass of appropriately trained teachers and a coincident critical mass of prospective students if delivered face to face.

### Building the critical mass of teachers and students

As a discipline, epidemiology may be able to draw on the model of the Biostatistics Collaboration of Australia http://www.bca.edu.au where there is cooperation from institutions in relation to reciprocal teaching arrangements and cross-institutional credit policies. The program is delivered by distance, thereby attracting a pool of students from across the country and now internationally. If institutions were to support a similar cooperative model for epidemiology, it could draw on the specific expertise of institutions (genetic epidemiology or the conduct of longitudinal studies, for example) without duplicating expertise in the delivery of short courses. This could ultimately act to increase access to a broad range of specialised training opportunities.

## Discussion

The key challenge for the workforce appears to be in raising the profile of epidemiological careers, creating pathways into specialised epidemiology training and extending existing training opportunities beyond MPH degrees in order to increase specialised epidemiology capacity in practitioners and researchers. The challenge is to increase the number of individuals who can operate effectively and who will be leaders within these career streams. Similarly, we must encourage more individuals who have the capacity to work across streams as researchers and/or practitioners, as it is likely that professional separation of practitioners and researchers in the past has contributed to the lack of clear career pathways in this discipline. Doing so will require ongoing discussion, cooperation and creative partnerships between academic institutions, government and employers.

The working group and workshops held were not structured to come up with an explicit set of recommendations about improving the epidemiology workforce. Nevertheless, there are several options for taking the issues discussed in this article forward. First, we acknowledge that a limitation of this article is the lack of evidence of a gap in epidemiology skills in Australia. Therefore, as a profession espousing evidence-based practice and policy, one of our first steps must be to investigate whether a gap in expertise exists, and avoid the pitfalls of relying on perceptions and anecdotal information!

Second, an updated formal epidemiology course survey, including specialised short courses, is a priority and will assist in exploring access issues and gaining a better understanding of the characteristics of the current student base. Third, the AEA is the most obvious group to formulate an action plan to address training issues. This is recognising that although the AEA membership represents only a small proportion of all individuals working in public health who practise epidemiology, it does include many senior epidemiologists responsible for epidemiology training in Australia.

Such an action plan could include strategies to: (a) identify and link the critical mass of practitioners and researchers available for teaching specialist epidemiology courses, and facilitate cross-institutional initiatives; and (b) facilitate dialogue between state-based services and academic institutions to optimise opportunities for joint and honorary appointments and thus research supervision. Other areas of action should include guidance on the definition of an epidemiology PhD and working with other professional bodies, such as the Australasian Faculty of Public Health Medicine, to reach agreement on core advanced competencies. Recommendations about critical conceptual understandings and advanced skills in the discipline may allow higher degree students and practitioners to more effectively seek out training in these competencies through coursework, applied short courses, short-term placements, or a combination of all three. Finally, the action plan may also encompass the development of a framework for benchmarking specialised epidemiology training programs. However, resource implications associated with this role need to be considered given the size and somewhat limited resources of the organisation.

More broadly, the organisation must expand its role in promoting epidemiology as a profession and providing a forum for discussion of workforce and other key issues for the discipline. Continuing an advocacy role in encouraging and supporting specific funding initiatives such as the National Health and Medical Research Council Capacity Building Grants for Population Health and Health Services Research is also essential to addressing financial constraints to building a career in epidemiology research.

## Conclusion

Although the extent of the epidemiology skills shortage in Australia remains to be quantified, participants in the AEA workshops and working group clearly saw opportunities for improvements in the formal education and training of epidemiologists. We have summarised the main strategies and educational models for increased access to specialised epidemiology training discussed in these forums; including conversion fellowships, formal integration of coursework into research higher degrees and specialised short courses.

This article is in no way intended to compete with work being undertaken by the PHERP and the Australian Network of Academic Public Health Institutions (ANAPHI) in developing standards for training in public health research and practice in Australia (as described in another article in this edition) [4]. Similarly, we do not wish to re-ignite the debate around accreditation of epidemiologists that dominated the council and annual general meetings of the AEA from its inception until 1999 [6]. We hope it will provoke greater public debate of these issues beyond the AEA membership, to facilitate input from new graduates through to senior researchers, trainers and practitioners. We now call on younger epidemiologists who feel they can fill the perceived gap in expertise to identify themselves, join AEA and take a stance on this issue, as did many of the now senior epidemiologists in the 1980s and early 1990s.

## Abbreviations

AEA: (Australasian Epidemiological Association); ANAPHI: (Australian Network of Academic Public Health Institutions); PHERP: (Public Health Education and Research Program).

## Competing interests

Both authors are involved in epidemiology teaching programs at their respective institutions.

## Authors' contributions

Both authors contributed equally and have read and approved the final manuscript.
